# COVID-19 vaccine uptake and associated factors among adolescents and youths: Findings and implications for future vaccination programmes

**DOI:** 10.1371/journal.pgph.0002385

**Published:** 2023-09-20

**Authors:** Steward Mudenda, Johanna C. Meyer, Joseph O. Fadare, Olayinka O. Ogunleye, Zikria Saleem, Scott K. Matafwali, Victor Daka, Billy Chabalenge, Jacob Chama, Moses Mukosha, Phumzile Skosana, Bwalya A. Witika, Aubrey C. Kalungia, Audrey Hamachila, Webrod Mufwambi, Brian Godman

**Affiliations:** 1 Department of Pharmacy, School of Health Sciences, University of Zambia, Lusaka, Zambia; 2 Department of Public Health Pharmacy and Management, School of Pharmacy, Sefako Makgatho Health Sciences University, Pretoria, South Africa; 3 South African Vaccination and Immunisation Centre, Sefako Makgatho Health Sciences University, Pretoria, South Africa; 4 Department of Pharmacology and Therapeutics, Ekiti State University, Ado Ekiti, Nigeria; 5 Department of Medicine, Ekiti State University Teaching Hospital, Ado Ekiti, Nigeria; 6 Department of Pharmacology, Therapeutics and Toxicology, Lagos State University College of Medicine, Ikeja, Lagos, Nigeria; 7 Department of Medicine, Lagos State University Teaching Hospital, Ikeja, Lagos, Nigeria; 8 Department of Pharmacy Practice, Faculty of Pharmacy, Bahauddin Zakariya University, Multan, Pakistan; 9 Clinical Research Department, Faculty of Infectious and Tropical Diseases, London School of Hygiene &Tropical Medicine, London, United Kingdom; 10 Department of Public Health, Michael Chilufya Sata School of Medicine, Copperbelt University, Ndola, Zambia; 11 Department of Medicines Control, Zambia Medicines Regulatory Authority, Lusaka, Zambia; 12 HIV and Women’s Health Research Group, University Teaching Hospital, Lusaka, Zambia; 13 Department of Clinical Pharmacy, School of Pharmacy, Sefako Makgatho Health Sciences University, Pretoria, South Africa; 14 Department of Pharmaceutical Sciences, School of Pharmacy, Sefako Makgatho Health Sciences University, Pretoria, South Africa; 15 Strathclyde Institute of Pharmacy and Biomedical Sciences, University of Strathclyde, Glasgow, United Kingdom; University of Embu, KENYA

## Abstract

Adolescents and youths are a key part of the population that needs to be protected against the coronavirus disease 2019 (COVID-19). This is because they are more likely to spread the virus to vulnerable individuals. In view of these concerns, this study investigated the uptake of COVID-19 vaccines and associated factors among adolescents and youths attending secondary schools in Zambia. This cross-sectional study was conducted among 1500 school-going adolescents in Lusaka from September 2022 to November 2022. Overall, 1409 participants took part giving a response rate of 94%. Only 29.2% (n = 411) of the participants were vaccinated against COVID-19 at the time of the study. Compared to their unvaccinated counterparts, vaccinated adolescents and youths scored higher for knowledge (66.2% vs 57.8%) and attitudes (76.7% vs 39.4%) regarding COVID-19 vaccines. Healthcare workers, family/friends and social media were key sources of information regarding the vaccine. Factors associated with increased vaccine uptake were positive attitudes (AOR = 33.62, 95% CI: 19.92–56.73), indicating it was stressful to follow COVID-19 preventive measures (AOR = 1.47, 95% CI: 1.09–1.99), participants in Grade 12 (AOR = 3.39, 95% CI: 1.94–5.91), Grade 11 (AOR = 2.59, 95% CI: 1.94–5.91), Grade 10 (AOR = 3.48, 95% CI: 1.98–6.11) and Grade 9 (AOR = 3.04, 95% CI: 1.74–5.32) compared to Grade 8. This study found a relatively low uptake of COVID-19 vaccines among adolescents and youths in Zambia. There is a need to provide adequate strategies to address knowledge and attitude gaps regarding COVID-19 vaccines to improve uptake and reduce future morbidity and mortality.

## Introduction

The coronavirus disease 2019 (COVID-19) pandemic remains a serious burden and priority globally increasing morbidity, mortality and costs including economic costs from lockdwn measures especially in low- and middle-income countries (LMICs) [1–3]. In addition, placing an unparalleled burden on the education sector across countries, especially during the earlier stages of the pandemic [4–8]. Before the development of effective vaccines and treatments, countries typically introduced a variety of public health preventative measures [9–12]. These included restrictions on gatherings, social distancing and lockdown measures incorporating the closure of borders, transport restrictions, closure of clinics as well as the mandatory wearing of face masks in public places [9–11, 13–16]. In many countries, lockdown measures also included the closure of classes with face-to-face learning in schools, colleges and universities [4–6, 17]. These measures were instigated in order to minimise the spread of infection among pupils, students, their teachers, and family members [17–20]. Despite these measures, COVID-19 continued to spread across countries, exacerbated not only by the variable implementation of public health measures but also concerns regarding their effectiveness [9, 12, 21, 22]. Consequently, there was an appreciable impetus globally to develop effective vaccines against COVID-19. One important impact in the case of children would be to restore routine immunisation programmes, which had been severely affected by the lockdown measures with considerable impact on future morbidity and mortality [23–25]. The primary goal of vaccination is to prevent pathogen-driven infections [26–29]. This maintains homeostasis by preventing infection-induced undesirable outcomes and training the immune system to re-program innate immune system cells to respond more effectively to future threats of the same antigenic type [30]. Their use to promote immunity and reduce the impact of infectious diseases has been reported in many areas and studies [30–35]. Following risk-benefit considerations, regulatory and medical decision-makers across countries sought to accelerate the development of COVID-19 vaccines to reduce the impact of the virus, which included the unintended consequences of lockdown and other measures [23, 36–38]. This feat was unprecedented considering vaccine development traditionally takes decades [39]. The rapid development of COVID-19 vaccines was made possible through previous research experience and tools supported by new manufacturing platforms, structure-based antigen design, computational biology, protein engineering and gene synthesis [39, 40].

The uptake of effective COVID-19 vaccines has varied across different populations and countries [41–47]. In general, COVID-19 vaccines have been well-received, with many people eager to be vaccinated in order to protect themselves and their communities from the virus [43, 48–51]. However, some groups across countries have been hesitant due to a variety of reasons, which have been fuelled by misinformation and/ or distrust of the vaccines [52–57]. The reasons for vaccine hesitancy are complex and vary across groups, time and regions [43, 46, 47, 58]. For example, surveys conducted among healthcare workers (HCWs) and students in various African countries found persistent vaccine hesitancy, which was principally due to concerns regarding their effectiveness and safety [47, 59–63]. However, higher acceptance rates have been reported among some European countries compared to the United States (US) and some African countries [43, 47, 51, 58, 64, 65].

Demographic and socio-economic characteristics also appear to influence the uptake of COVID-19 vaccines. Certain racial and ethnic groups, alongside lower-educated and lower-income people, are typically less likely to receive vaccines. This is potentially due to a lack of access as well as longstanding mistrust of the medical system [43, 66, 67]. Public health officials need to address these disparities, alongside ongoing misinformation, to ensure vaccines are readily available, accessible, and administered to all members of society to maximise the benefit of vaccines [47].

Children, adolescents, and youths attending school are key among the vulnerable populations at risk of contracting COVID-19 and subsequently spreading the virus, especially as vaccination in populations aged 12 to 17 years has only recently been approved, with hospitalisation rates increasing in countries pre-vaccination [68, 69]. To date over 17,000 children worldwide have died from COVID-19, and in the USA, COVID-19 was the seventh leading cause of death among children aged 5–11 years, and even higher for children aged 12–18 years leading to calls to vaccinate all children above the age of 5 [70, 71]. They also remain a key group for herd immunity, and encouraging vaccination in this population will help protect children and the community in the future [72]. There are also growing concerns about possibly increased mortality in this group in LMICs, enhanced by new variants [73, 74]. In addition, there is currently overuse of antibiotics when children with actual or suspected COVID-19 are admitted to the hospital, increasing antimicrobial resistance (AMR) [75–78]. AMR is a key concern in sub-Saharan Africa with high and growing rates, further increasing morbidity, mortality and costs [79–81].

Consequently, vaccination against COVID-19 is of increasing importance in this population to protect them from severe disease, including subsequent hospital admission, if contracted [68, 72, 82, 83]. However, vaccine hesitancy has been reported among adolescents in sub-Saharan Africa [84]. This is a concern as low acceptance of the COVID-19 vaccines may eventually lead to low uptake as seen with other vaccines [85, 86]. In addition, low uptake of this vaccine may reduce the uptake of other vaccines against infectious diseases in Africa, with vaccination rates already severely affected by lockdown and other measures in the early stages of the pandemic [23, 24].

A low acceptance rate of COVID-19 vaccines was reported among pupils attending secondary schools in Zambia, despite most of them having good knowledge and attitudes towards the vaccine [87]. However, there is currently insufficient information regarding the actual uptake of COVID-19 vaccines among adolescents and youths attending secondary schools in Zambia to further refine policies to improve future vaccination rates in this critical group. We also believe it is critical to compare and contrast attitudes towards the vaccine among both vaccinated and unvaccinated populations to provide future guidance to key stakeholders in Zambia if concerns persist. Alongside this, the Zambian government recently (January 2022) commenced COVID-19 vaccination for all children from the age of 12 to 17 years [47], with 222,300 doses Pfizer-BioNTech vaccine being the first to be received for this role [88]. Consequently, this study investigated the uptake of COVID-19 vaccines and associated factors among adolescents and youths attending secondary schools in Lusaka, Zambia. The findings from this comprehensive approach can be used to develop more robust age-appropriate interventions to promote confidence in the vaccines in this important sub-population in Zambia and beyond given the ongoing public health concerns.

## Materials and methods

### Study design, setting and population

This cross-sectional study involved both vaccinated and unvaccinated pupils aged 12 years and older attending secondary schools in Lusaka. Lusaka was chosen for this study because it is Zambia’s capital city and the country’s first COVID-19 epicentre [89, 90]. The findings will help determine the COVID-19 vaccine uptake among the secondary school population in Lusaka city, and not just intentions, and to more comprehensively explore potential associations between vaccination uptake and participants’ knowledge and attitudes. Secondary schools in Zambia provide education (Levels: Grade 8 to Grade 12) to adolescents (defined as persons aged 10 to 19 years) and youths (defined as persons aged 15 to 35 years) [91]. We appreciate that up to 35 years is typically seen as old for youths; however, this is the current situation in Zambia.

### Sampling method and sample size calculation

Multi-stage sampling was used to select 32 out of 111 secondary schools in Lusaka city, representative of the four sub-districts, with two to four classes selected, randomly and proportional to the school size [87]. Participants from the selected classes were recruited using simple random sampling (computer-generated random numbers without replacement). The effective sample size for a single population was estimated at 543, using the Raosoft software http://www.raosoft.com/samplesize.html (accessed on 22 July 2022). A 10% provision was made for possible losses, incomplete, or non-responses and a design effect of 1.5 was used to account for the loss in precision due to clustering.

### Data collection process and tool

A pre-validated, self-administered questionnaire was used to collect data [87]. The questionnaire consisted of five parts, collecting data on the following components: i) Sociodemographic characteristics of the students; ii) their knowledge of COVID-19 vaccines; iii) Attitudes towards COVID-19; iv) Acceptance and uptake of COVID-19 vaccines; v) and key factors that could influence the acceptance and uptake of COVID-19 vaccines (S1 Text).

A pilot study was undertaken before the full study to check for the feasibility and simplicity of the questionnaire to enhance its robustness. No changes were subsequently made to the questionnaire. The final questionnaire had acceptable reliability (Cronbach’s alpha of 0.842 for attitude and 0.762 for knowledge) and required 20 to 30 minutes to complete. The findings from the pilot study were not included in the main study findings and statistical analysis. Data regarding the vaccinated and unvaccinated participants were collected between September 2022 and November 2022 by two trained data collectors. Participants were first informed about the study. Upon providing written assent from the youths or obtaining written consent from the parents and/or guardians of adolescents, the questionnaire was subsequently distributed to the adolescents and youths attending the selected secondary schools. The findings were subsequently compared to the previous study on intentions to vaccinate against COVID-19 to help formulate pertinent strategies to improve vaccination rates in this population where concerns were identified [87].

### Data management and analysis

Data were entered and cleaned for consistency and range checks in Microsoft Excel® (Redmond, WA) (S1 Data). The data were subsequently exported to Stata® version 17/BE (Stata Corp., College Station, Texas, USA) for statistical analysis. Knowledge and attitude questions were assigned a score of one for “Yes” responses and a zero for “No or don’t know” responses. The item scores were summed to create a total score for knowledge and attitude. Descriptive analyses were subsequently performed to assess knowledge and attitude scores for vaccinated participants.

Robust estimations of standard errors were used to account for the clustering of participants within schools in all analyses. Clopper-Pearson’s exact method was subsequently used to calculate the 95% binomial confidence interval (CI) of the overall proportion of vaccinated pupils. Following this, we fitted univariable logistic regression models with robust estimation of standard errors with ‘COVID-19 vaccine uptake’ as the outcome variable and one of the predictor variables at a time, to assess evidence of an association with COVID-19 vaccine uptake by assessing crude odds ratios (CORs). Subsequently, a multiple logistic regression model was fitted with variables that were significant in the univariable model at p<0.2 using investigator-led stepwise regression, with only knowledge and attitudes set as priori variables.

Interactions between significant predictors in the final model were subsequently considered; however, none reached statistical significance considered as p≤0.05. Finally, adjusted odds ratios (AORs) at 95% CI were used to identify statistically significant predictors of COVID-19 vaccine uptake. The goodness of fit of the model was assessed using the Hosmer-Lemeshow test.

### Ethical approval

Ethical approval was granted by the University of Zambia Health Sciences Research Ethics Committee (UNZAHSREC). The study was approved under the protocol ID: 202211231183, IORG no: 0009227, IRB no: 00011000. Clearance to collect data was also obtained from the Lusaka District Education Board (DEB) and the management of the selected schools. We informed the participants of the purpose of this study and they assented to be part of the study. Additionally, we obtained consent from the participant’s parents or guardians for the adolescents to participate in the study. The youths provided consent on their own to be part of the study.

### Inclusivity in global research

Additional information regarding the ethical, cultural, and scientific considerations specific to inclusivity in global research is included in the S2 Text.

## Results

### Response rate

In total, 1500 questionnaires were distributed to the combined study population, of which 1409 completed questionnaires were returned (94% response rate). The final sample size provided more than 80% power to detect the minimum difference in the rates of COVID-19 vaccine uptake among secondary school-attending adolescents and youths with precision ±5% and 95% CIs.

### COVID-19 vaccine uptake, sociodemographic characteristics, knowledge and attitudes

Overall, 29.2% (95% CI: 26.8%-31.6%) of participants had received at least one dose of the COVID-19 vaccine. The mean age of the participants was 15.8 years [Standard Deviation (SD) = 1.9], ranging from 12 to 26 years. The largest proportion of participants (n = 929, 65.9%) were females, 1336 (94.8%) were living at home with their parents and 363(25.8%) were in Grade 11 (Table 1).

**Table 1:** Sociodemographics, knowledge and attitude scores distributed by COVID-19 vaccine uptake.

Variables		Total population; n (%) (N = 1409)	Vaccinated against COVID-19; n (%)	
			No (n = 998)	Yes (n = 411)
**Sex**	Female	929(65.9)	646(64.7)	283(68.9)
	Male	480(34.1)	352(35.3)	128(31.1)
**Living with**	Guardian	73(5.2)	43(4.3)	30(7.3)
	Parents	1336(94.8)	955(95.7)	381(92.7)
**School Level**	Grade 8	222(15.7)	185(18.5)	37(9.0)
	Grade 9	281(19.9)	200(20.0)	81(19.7)
	Grade 10	253(17.9)	163(16.3)	90(21.9)
	Grade 11	363(25.8)	252(25.3)	111(27.0)
	Grade 12	290(20.5)	198(19.8)	92(22.4)
**Source of information about COVID-19**				
Healthcare workers	No	912(64.7)	651(65.2)	261(63.5)
	Yes	497(35.3)	347(34.8)	150(36.5)
Mass Media (Television/ Radio)	No	676(48.0)	469(47.0)	207(50.4)
	Yes	733(52.0)	529(53.0)	204(49.6)
Social Media	No	933(66.2)	635(63.6)	298(72.5)
	Yes	476(33.8)	363(36.4)	113(27.5)
Family/ Friends	No	1065(75.6)	749(75.1)	316(76.9)
	Yes	344(24.4)	249(25.0)	95(23.1)
**Key characteristics**				
Age (years)	Mean (SD)	15.8(1.9)	15.6(3.6)	16.1(1.9)
Knowledge Score	Mean % (SD)	60.3(28.4)	57.8(28.1)	66.2(27.0)
Attitude Score	Mean % (SD)	50.3(37.2)	39.4(34.3)	76.7(30.0)

Approximately three in five participants reported HCWs, family/friends and social media as the principal sources of information regarding the COVID-19 pandemic. Overall, participants reported a mean COVID-19 vaccine knowledge and attitude percentage score of 60.3% (SD = 28.4) and 50.3% (SD = 37.2), respectively. Compared to participants who were not vaccinated, the vaccinated participants had greater knowledge (66.2% vs 57.8%) and attitude (76.7% vs 39.4%) scores regarding the COVID-19 vaccine (Table 1).

### COVID-19 experience

The majority (n = 1187, 84.2%) of the participants had not suffered from COVID-19, did not know a relative/friend who died from COVID-19 (n = 1009, 71.6%) and had never been quarantined due to COVID-19 (n = 991, 70.3%).

Approximately half (n = 680, 48.3%) of the participants knew a relative/friend who suffered from COVID-19 disease, and just over half (n = 765, 54.3%) reported that preventive measures were not stressful to follow. A large proportion of participants (n = 1087, 77.2%) were able to practice social distancing, and an appreciable number (n = 1244, 88.3%) had never suffered from a chronic condition (Table 2).

**Table 2:** COVID-19 experiences of participants according to COVID-19 vaccine uptake.

Experiences/condition		Total population; n (%) (N = 1409)	Vaccinated against COVID-19; n (%)	
			No (n = 998)	Yes (n = 411)
Suffered from COVID-19 before	I don’t know	74(5.3)	45(4.5)	29(7.1)
	No	1187(84.2)	855(85.7)	332(80.8)
	Yes	148(10.5)	98(9.8)	50(12.2)
Relatives/Friends suffered from COVID-19	I don’t know	92(6.5)	60(6.0)	32(7.8)
	No	637(45.2)	471(47.2)	166(40.4)
	Yes	680(48.3)	467(46.8)	213(51.8)
Relatives/Friends died from COVID-19	I don’t know	151(10.7)	107(10.7)	44(10.7)
	No	1009(71.6)	733(73.5)	276(67.2)
	Yes	249(17.7)	158(15.8)	91(22.1)
Quarantined as a result of COVID-19	I don’t know	128(9.1)	95(9.5)	33(8.0)
	No	991(70.3)	710(71.1)	281(68.4)
	Yes	290(20.6)	193(19.3)	97(23.6)
Able to practice physical and social distancing	I don’t know	88(6.3)	52(5.2)	36(8.8)
	No	234(16.6)	167(16.7)	67(16.3)
	Yes	1087(77.2)	779(78.1)	308(74.9)
Preventive measures are stressful to follow	I don’t know	132(9.4)	85(8.5)	47(11.4)
	No	765(54.3)	558(55.9)	207(50.4)
	Yes	512(36.3)	355(35.6)	157(38.2)
Suffering from a chronic condition	I don’t know	79(5.6)	52(5.2)	27(6.6)
	No	1244(88.3)	888(89.0)	356(86.6)
	Yes	86(6.1)	58(5.8)	28(6.8)

### Predictors of COVID-19 vaccine uptake

The multivariable logistic regression model showed that independent factors associated with COVID-19 vaccine uptake were attitude, school grade and COVID-19 preventive measures being stressful to follow (Table 3).

**Table 3:** Predictors of COVID-19 vaccine uptake among children, adolescents, and youths.

Characteristics		COR (95% CI)	p-value	AOR (95% CI)	p-value
Attitude score		25.87(16.83–39.76)	<0.001	33.62(19.92–56.73)	<0.001
Knowledge score		3.03(1.96–4.70)	<0.001	1.05(0.60–1.83)	0.873
Socio demographics					
Age (years)		1.13(1.07–1.20)	<0.001		
Sex	Female	Ref			
	Male	0.83(0.65–1.06)	0.138		
Living with	Guardian	Ref		Ref	
	Parents	0.57(0.35–0.93)	0.023	0.53(0.27–1.03)	0.062
School level	Grade 8	Ref		Ref	
	Grade 9	2.03(1.31–3.14)	0.002	3.04(1.74–5.32)	<0.001
	Grade 10	2.76(1.78–4.27)	<0.001	3.48(1.98–6.11)	<0.001
	Grade 11	2.20(1.45–3.34)	<0.001	2.59(1.94–5.91)	<0.001
	Grade 12	2.32(1.51–3.58)	<0.001	3.39(1.94–5.91)	<0.001
**Source of information about COVID-19**					
Healthcare workers	No	Ref			
	Yes	1.08(0.85–1.37)	0.538		
Mass media (TV/radio)	No	Ref			
	Yes	0.87(0.69–1.10)	0.250		
Social Media	No	Ref			
	Yes	0.66(0.52–0.85)	0.001		
Family/friends	No	Ref			
	Yes	0.90(0.69–1.19)	0.466		
**COVID-19 experiences**					
Suffered from COVID-19 before	No	Ref			
	Yes	1.31(0.91–1.89)	0.141		
Relatives/Friends suffered from COVID-19		Ref		Ref	
		1.29(1.02–1.65)	0.035	1.32(0.99–1.78)	0.062
Relatives/Friends died from COVID-19	No	Ref			
	Yes	1.53(1.14–2.05)	0.004		
Quarantined as a result of COVID-19	No	Ref			
	Yes	1.27(0.96–1.68)	0.095		
Able to practice physical and social distancing	No	Ref			
	Yes	0.98(0.72–1.35)	0.927		
Preventive measures are stressful to follow	No	Ref		Ref	
	Yes	1.19(0.93–1.53)	0.162	1.47(1.09–1.99)	0.012
Suffering from a chronic condition	No	Ref			
	Yes	1.20(0.75–1.92)	0.436		

Participants with higher attitude scores (AOR = 33.62, 95% CI: 19.92–56.73) and those who reported that COVID-19 preventive measures were stressful to follow (AOR = 1.47, 95% CI: 1.09–1.99) were more likely to be vaccinated against COVID 19. In addition, participants who were in Grade 12 (AOR = 3.39, 95% CI: 1.94–5.91), Grade 11 (AOR = 2.59, 95% CI: 1.94–5.91), Grade 10 (AOR = 3.48, 95% CI: 1.98–6.11) or Grade 9 (AOR = 3.04, 95% CI: 1.74–5.32) were more likely to be vaccinated against COVID 19 versus those in Grade 8 (Table 3).

## Discussion

We believe this is the first study in Zambia to investigate the current uptake of COVID-19 vaccines among adolescents and youths attending secondary schools, where the national mass COVID-19 vaccine administration program is taking place in the country. Overall, the vaccine uptake among this population at the time of the study was 29.2% (n = 411). The vaccine uptake was higher among participants with better COVID-19 knowledge and attitude scores as well as higher school grades. Encouragingly, the actual uptake of the COVID-19 vaccine was higher than the findings of an earlier study among unvaccinated school children and adolescents in Zambia where only 12.7% of participants indicated that they would accept to be vaccinated with COVID-19 vaccines [87]. This shows the benefit of conducting studies not only about intentions but also about subsequent actions to guide future strategies.

However, the low uptake of COVID-19 vaccines among school-going adolescents and youths in our study is a public health concern. Having said this, the uptake rate was similar to the findings from a multi-country study conducted in the Eastern Mediterranean region at 32% [92], and in the US where only 25.6% of the adolescents in the study were vaccinated [93]. This though has increased in the US in recent months [69, 94]. We are also aware in the study of Wang et al. (2022) that only 12% to 14% of adolescents in Tanzania were willing to be vaccinated, and only 35% of adolescents in Nigeria [84]. These combined findings could be due to limited knowledge and negative attitudes among school adolescents and youths regarding COVID-19 vaccines. Alongside this, the overall attitude of adult residents in their communities regarding COVID-19 vaccination may have some influence on our study population. This is a concern as low uptake of COVID-19 vaccines can affect national goals of protecting the majority of school-going children, adolescents and youths against the coronavirus and its effects.

Encouragingly, a recent study in Italy reported a vaccination rate of 87.3% among school-going adolescents [95]. Additionally, in Turkey, the vaccination rate among youths was 80% [96]. Alongside this in Kersa, Ethiopia, 86% of adolescents stated they would seek vaccination once available [84]. In Turkey, the youths wanted to be vaccinated to protect their health as well as the health of their families and relatives [96]. In Brazil, a recent study reported that 88.7% of caregivers wanted their children to be vaccinated while 77.6% of parents in China wanted their children to be vaccinated against COVID-19 [97, 98].

In the US, the authors found that parental confidence in the COVID-19 vaccines was critical to increasing the number of children and adolescents vaccinated [69]. This is similar to other countries and other vaccines for children, including HPV vaccines [84, 92, 99–101]. These findings indicate a key role of parents and caregivers in improving the acceptance and uptake of COVID-19 vaccines among children and adolescents.

The main sources of information on COVID-19 among the study participants were mass media, HCWs, and social media. Others have also reported that these sources of information are influential in spreading correct information and misinformation about COVID-19 vaccines [56, 102–107]. Similarly in Taiwan, among university students in Jordan, and among adolescents across Africa, the internet and social media were the most common sources of information regarding COVID-19 and its vaccines [84, 108, 109]. This shows that mass media should increasingly be used as a platform for sharing credible information about COVID-19 vaccines and their benefits as well as addressing misinformation to reduce the morbidity, mortality and costs associated with COVID-19 [110, 111].

Concerns regarding the effectiveness and safety of COVID-19 vaccines among HCWs need also to be addressed going forward as HCWs play a critical role in sharing information about COVID-19 vaccines [47, 112–115]. Traditional sources of information such as television and radio have also been used as key sources of information on COVID-19 and its vaccines in many settings [116–118]; however, their role is changing in favour of social media and other platforms. This needs to be taken into consideration when health authorities and Governments are developing and refining their strategies to address vaccine hesitancy generally.

We found that most of the vaccinated adolescents and youths in our study had relatives or friends that had suffered or died from COVID-19. This suggests that individuals who have been directly impacted by the virus due to sickness or the death of loved ones may be more inclined to seek vaccination as a means of protecting themselves and family members, which is similar to other studies [92, 115, 119–121]. Public health campaigns need to recognise and address this motivation in order to effectively promote vaccination and mitigate the spread of COVID-19 amongst this key group.

Additionally, we found that some of the vaccinated participants had previously been quarantined due to COVID-19 exposure or had a pre-existing chronic condition. This suggests that individuals who are at higher risk for severe illness from COVID-19, especially those with co-morbidities, may be more likely to prioritise vaccination to protect their health, again similar to other studies [120, 122–125]. Consequently, future public health campaigns need to consider these additional risk factors as well and target outreach and education efforts towards individuals who may be more vulnerable to severe COVID-19 illness. Together with this, parents alongside key HCWs are important targets for vaccinating children, adolescents, and youths.

We also found that participants who had higher odds of being vaccinated were in higher grades (Grade 9, -10, -11, and -12 compared to Grade 8). In addition, those that found prevention measures stressful to follow had higher odds of being vaccinated, with a likely reduction in preventative measures as more of the population become vaccinated. These findings imply that promoting vaccine uptake can be achieved by targeting appropriate socio-demographic populations and those with negative attitudes and poor knowledge concerning COVID-19 vaccines [126–128]. This is also evidenced by previous studies that have shown that parents’ and caregivers’ positive attitudes can promote vaccine acceptance and uptake[121, 129–132]. This further emphasises the role of governments, HCWs, teachers and other stakeholders in promoting positive messages regarding the positive effects of COVID-19 vaccines among school children, adolescents and youths as well as adults to counteract ongoing misinformation.

We are aware of a number of limitations of this study. Firstly, this study was only conducted in a population of adolescents and youths attending secondary schools in Lusaka, hence, generalisation of the findings should be done with caution. Consequently, future studies should investigate the entire population of schooling and non-schooling adolescents and youths in Zambia. Secondly, this was a survey as opposed to an in-depth qualitative discussion with participants. Thirdly, being a cross-sectional study in nature, our findings may be affected by recall bias. Finally, we did not ask for the name of the COVID-19 vaccine that each vaccinated participant had received. However, despite this, we consider our findings robust given the choice of the setting and the extensive methodology involved. Future studies can be conducted to investigate further factors that contribute to vaccine hesitancy among schooling and non-schooling adolescents and youths in Zambia as the roll-out of the vaccine continues in this younger age group. The findings can subsequently be used to develop future strategies, including social media strategies, to enhance vaccine acceptance and uptake in this critical population, with implications for other infectious diseases.

## Conclusion

We found a relatively low uptake of the COVID-19 vaccines among adolescents and youths attending secondary schools in Zambia. The low uptake of the COVID-19 vaccine generally among adolescents and youth is a public health concern as this may hinder the goals of vaccination programmes against COVID-19 as well as generally among this population. Consequently, there is a need to develop mainstream youth-friendly educational interventions and associated communication programs to enhance the acceptance and uptake of COVID-19 vaccines in adolescents, and youths. Further studies should be undertaken to monitor this.

## Supporting information

S1 Text Study questionnaire. (DOC)

S1 Checklist STROBE statement—checklist of items that should be included in reports of observational studies. (DOCX)

S1 Data Study dataset. (XLSX)

S2 Text Plos GPH questionnaire. (DOCX)
