# Supplementary material for: COVID-19 vaccine uptake and associated factors among adolescents and youths: Findings and implications for future vaccination programmes
Source: PLOS Glob Public Health. 2023 Sep 20;3(9):e0002385. doi: 10.1371/journal.pgph.0002385 (PMC10511127; doi:10.1371/journal.pgph.0002385)
Supplement: S1 Text — (DOC) [file pgph.0002385.s001.doc]

Uptake of COVID-19 Vaccines among Adolescents and Youths in Zambia

*COVID-19 vaccine uptake*

*Page 1*

| **Part I**: **Sociodemographic characteristics of pupils** | | |
| --- | --- | --- |
| SN | **Questions** | **Answers/choice** |
| 1 | Age | years |
| 2 | Grade |  |
| 3 | School |  |
| 4 | Gender | 1. Male  2. Female |
| 5 | Whom do you live with? | 1. Parents  2. Guardians |
| **Part II: Knowledge questions about COVID -19 vaccine [circle the correct answer]** | | |
| 1 | Have you ever heard about the COVID-19 vaccine? | 1. Yes 2.No |
| 2 | If said "yes "to question 1 above, what is the main source of information? | 1. Healthcare workers  2. TV/radio  3. Social media  4. Family/friends  5. Others….. |
| 3 | Do you know currently people taking the COVID-19 vaccine? | 1. Yes 2.No 3. I don't know |
| 4 | Do you think COVID-19 vaccines reduce disease transmission? | 1. Yes 2.No 3. I don't know |
| 5 | Do you think the use of COVID-19 vaccines has side effects? | 1. Yes 2.No 3. I don't know |
| **Part III: Attitude questions about the COVID-19 vaccine** | | |
| 1 | Taking the COVID-19 vaccine is important for our health. | 1. Yes 2.No 3. I don't know |
| 2 | I will take the COVID-19 vaccine without any fear. | 1. Yes 2.No 3. I don't know |
| 3 | I will encourage my family /friends to take the COVID-19 vaccine. | 1. Yes 2.No 3. I don't know |
| 4 | COVID-19 vaccine should be distributed fairly to all of us. | 1. Yes 2.No 3. I don't know |
| 5 | I support currently recommended COVID-19 vaccine campaigns and programs. | 1. Yes 2.No 3. I don't know |
| **Part IV: Uptake of COVID-19 vaccines** | | |
| 1 | Have you been vaccinated against COVID-19? | 1. Yes 2. No |
| 2 | Would you accept to be vaccinated against COVID-19? | 1. Yes 2. No 3. I don’t know |
| **Part V: Factors influencing participants’ acceptance of COVID-19 vaccines** | | |
| 1 | Have you ever suffered from COVID-19? | 1. Yes 2.No 3. I don't know |
| 2 | Did any of your friends or relatives suffer from COVID-19? | 1. Yes 2.No 3. I don't know |
| 3 | Has any of your friends or relatives died from COVID-19? | 1. Yes 2.No 3. I don't know |
| 4 | Were you ever quarantined as a result of COVID-19? | 1. Yes 2.No 3. I don't know |
| 5 | Are you able to practice physical and social distancing? | 1. Yes 2.No 3. I don't know |
| 6 | Are the preventive measures of COVID-19 stressful to follow? | 1. Yes 2.No 3. I don't know |
| 7 | Do you suffer from ANY chronic condition (tuberculosis, diabetes mellitus, HIV/AIDs, asthma, bronchitis, hypertension, cancer)? | 1. Yes 2.No 3. I don't know |

Thank you for your participation
